# Hypertrophic Cardiomyopathy with Special Focus on Mavacamten and Its Future in Cardiology

**DOI:** 10.3390/biomedicines12122675

**Published:** 2024-11-24

**Authors:** Ewelina Młynarska, Ewa Radzioch, Bartłomiej Dąbek, Klaudia Leszto, Alicja Witkowska, Witold Czarnik, Weronika Jędraszak, Jacek Rysz, Beata Franczyk

**Affiliations:** 1 Department of Nephrocardiology, Medical University of Lodz, ul. Zeromskiego 113, 90-549 Lodz, Poland; 2 Department of Nephrology, Hypertension and Family Medicine, Medical University of Lodz, ul. Zeromskiego 113, 90-549 Lodz, Poland

**Keywords:** cardiomyopathies, hypertrophic cardiomyopathy, mavacamten, cardiac therapeutics

## Abstract

Hypertrophic cardiomyopathy (HCM) is a heterogeneous group of heart muscle disorders that affects millions, with an incidence from 1 in 500 to 1 in 200. Factors such as genetics, age, gender, comorbidities, and environmental factors may contribute to the course of this disease. Diagnosis of HCM has improved significantly in the past few decades from simple echocardiographic evaluations to a more complex, multimodal approach embracing advanced imaging, genetic, and biomarker studies. This review focuses on Mavacamten, a selective allosteric inhibitor of cardiac myosin, as a pharmacological treatment for HCM. Patients with HCM experience pathological actomyosin interactions, leading to impaired relaxation and increased energy expenditure. Mavacamten decreases available myosin heads, reducing actomyosin cross-bridges during systole and diastole. By reducing the number of bridges left ventricular outflow tract pressure is normalized and cardiac cavities are filled. This mechanism enhances patient performance and alleviates symptoms such as chest pain and dyspnea. The results suggest the potential for Mavacamten to transform the treatment of obstructive hypertrophic cardiomyopathy. Studies to date have shown significant improvement in exercise capacity, symptom relief, and a reduction in the need for invasive procedures such as septal myectomy. Further studies are needed to confirm the clinical results.

## 1. Introduction

Cardiomyopathies constitute a heterogeneous group of disorders affecting the heart muscle, characterized by structural and functional abnormalities that cannot be fully explained by the presence of coronary artery disease, hypertension, valvular diseases, or congenital heart defects [1,2,3]. These disorders may have both hereditary and acquired origins and present with a diverse clinical course, ranging from asymptomatic forms to severe cases, manifesting as heart failure, malignant arrhythmias, or sudden cardiac death [2].

**Figure 1:**
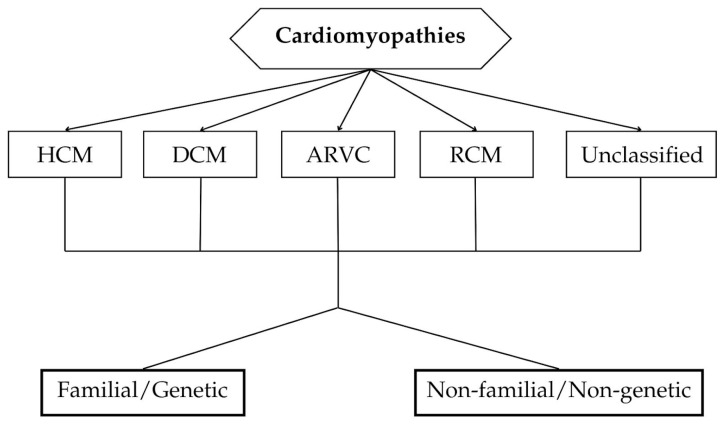
Summary of the proposed cardiomyopathy classification system [1]. ARVC indicates arrhythmogenic right ventricular cardiomyopathy; DCM, dilated cardiomyopathy; HCM, hypertrophic cardiomyopathy; and RCM, restrictive cardiomyopathy.

From a morphofunctional phenotypic perspective, cardiomyopathies are classified into hypertrophic cardiomyopathy (HCM), dilated cardiomyopathy (DCM), restrictive cardiomyopathy (RCM), arrhythmogenic right ventricular cardiomyopathy (ARVC), and unclassified cardiomyopathy [3,4]. The contemporary approach to cardiomyopathy classification, which is based on morphological and functional criteria, does not fully encompass the underlying etiology of these conditions. Furthermore, each subtype has distinct pathophysiology, clinical presentation, and disease progression, underscoring the need for further subdivisions within this group of disorders [3,4]. The classification proposed by the American Heart Association (AHA) categorizes cardiomyopathies into genetic, mixed, and acquired forms, while the European Society of Cardiology (ESC) suggests an additional subdivision of each major category into familial or genetic forms and non-familial or non-genetic forms (Figure 1) [1,3]. In this context, “familial” refers to the presence of the same disorder or phenotype in more than one family member, typically due to the same genetic mutation. This definition does not apply to acquired heart diseases or systemic disorders, where the clinical phenotype is influenced by genetic polymorphisms. Most familial cardiomyopathies are classified as monogenic disorders, which can occur sporadically when the causal mutation is de novo. Patients with identified de novo mutations are assigned to the familial category, as their disorder may be transmitted to offspring. Conversely, non-familial cardiomyopathies are defined by the occurrence of the condition in an index patient, with no other affected family members, as confirmed by pedigree analysis and clinical assessment. These are further divided into idiopathic cardiomyopathies, where the cause is unknown, and acquired forms, where chamber dysfunction is a complication related to another condition rather than an intrinsic feature of the disease itself [4].

The ESC classification is crucial for distinguishing different types of cardiomyopathies, which are highly heterogeneous from a clinical standpoint, with genotype being one of the differentiating factors. In this review, we will focus on the most common hereditary cardiomyopathy, namely HCM [1,2,3,4,5], which results from mutations in various genes. Understanding the relationship between genotype and phenotype and exploring the mechanisms underlying the development of cardiomyopathy based on causative genes, are key to developing new therapeutic strategies. Over the past decade, significant advancements have been made in the diagnosis and treatment of HCM [6]. Emerging therapies, such as drugs targeting specific signaling pathways within the myocardium, offer the potential to alter the natural course of HCM, improving patient outcomes and reducing the risk of complications. Therefore, this review aims to provide a comprehensive analysis of the current therapeutic options for HCM, with a specific focus on the potential of Mavacamten as an innovative treatment. It examines the pathophysiological mechanisms underlying HCM, recent advancements in diagnostic approaches, and the potential of Mavacamten, a selective allosteric inhibitor of cardiac myosin, to improve patient outcomes. Furthermore, the article highlights significant findings from clinical trials, demonstrating the efficacy of Mavacamten in alleviating symptoms and enhancing exercise capacity.

## 2. Pathogenesis of HCM

**Figure 2:**
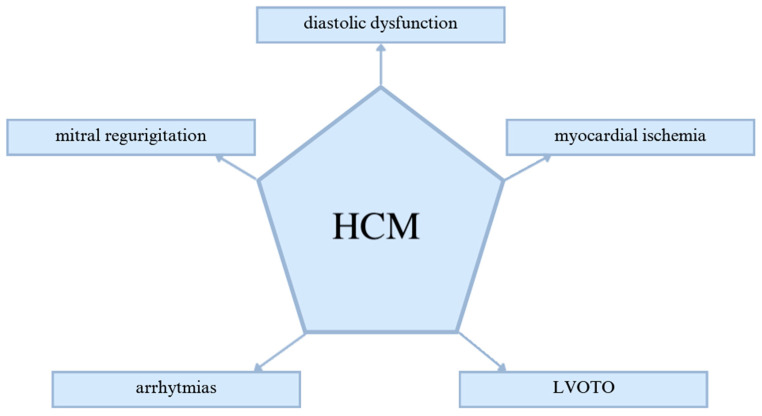
Pathophysiological changes in hypertrophic cardiomyopathy [11]. LVOTO indicates left ventricular outflow track obstruction; HCM, hypertrophic cardiomyopathy.

HCM is a classic example of a monogenic disorder inherited in an autosomal dominant manner [7]. Most patients with HCM are heterozygous for truncating or missense mutations in genes encoding sarcomeric proteins, primarily cardiac myosin-binding protein-C (MYBPC3), β-myosin heavy chain (MYH7), and troponin T2 (TNNT2) [8,9,10,11,12]. In the course of HCM, a series of pathophysiological changes arise, as illustrated in Figure 2 [11].

The result of genetic mutations responsible for HCM is the expression of abnormal proteins. The incorporation of these pathological proteins into the sarcomere leads to a range of structural and functional abnormalities within the sarcomere. The primary function of the sarcomere is force generation, which occurs through the bending of the myosin globular head at the hinge region and the subsequent displacement of the actin filament [13]. The mechanism by which mutations induce functional alterations primarily involves changes in Ca^2+^ sensitivity and ATPase activity. For example, mutations in MYBPC3 and MYH7 negatively impact force generation by altering ATPase activity. Notably, MYH7 mutations reduce myofibrillar ATPase activity more significantly than those in MYBPC3 [14,15]. Consequently, the energetic cost of force production is substantially higher in MYH7 mutations compared to MYBPC3 mutations. The increased energy cost of tension generation is associated with a reduced ratio of cardiac phosphocreatine to ATP in the heart in HCM [16]. A 2017 meta-analysis indicates that the mean septal thickness is higher among patients with HCM who carry mutations in the *MYBPC3* and *MYH7* genes compared to those with *TNNT2* mutations or patients without detectable mutations. Additionally, patients with *MYH7* mutations are more likely to manifest the disease and tend to experience more severe symptoms than those with *MYBPC3*-related HCM. Furthermore, *MYH7*-associated HCM is more frequently linked to progression to end-stage heart failure, with a higher requirement for heart transplantation (HTx) compared to *MYBPC3* HCM cases [17].

Structural and functional changes in the sarcomere trigger a cascade of stress-responsive molecular pathways, ultimately leading to histological and morphological alterations in cardiac muscle. Several pathways become dysregulated, including the activation of mitotic and trophic factors, such as the transforming growth factor-beta (TGF-β) pathway, calcineurin, and mitogen-activated protein kinases (MAPKs) [13,18]. The activation of these intermediary molecules and pathways results in interstitial fibrosis, cardiac hypertrophy, and myocyte disarray, which contribute to various disturbances in cardiac function [19,20].

Cardiac hypertrophy in HCM is often asymmetrical and, in most cases, primarily involves the basal interventricular septum beneath the aortic valve, as well as the free wall of the left ventricle (LV). The typical ratio of septal thickness to the free wall of the ventricle is ≥1.3:1. In some cases, the hypertrophy is predominantly localized to the cardiac apex, a variant referred to as “apical HCM”. In a smaller number of cases, hypertrophy is primarily found in the lateral or posterior walls of the heart.

The aforementioned hypertrophy, along with fibrosis of the cardiac muscle, leads to chamber reduction and stiffening, thereby impairing both systolic and diastolic function [13,21,22]. Septal hypertrophy leads to narrowing of the left ventricular outflow tract (LVOT), obstructing blood flow, which subsequently results in anterior displacement of the mitral valve leaflets, as well as anatomical distortions of the mitral valve apparatus, such as elongated leaflets and displacement of the papillary muscles. Pathological changes in the mitral valve lead to mitral regurgitation, left ventricular outflow tract obstruction (LVOTO), and increased pressure within the LV. This results in elevated systemic pressure, which exacerbates left ventricle hypertrophy (LVH), myocardial ischemia, and prolongation of ventricular relaxation, further worsening diastolic dysfunction [23,24]. Impaired stroke volume and an increased risk of heart failure (HF) are strongly associated with LVOTO. Even minor changes in preload or afterload, or an increase in contractility, can lead to a rise in the LVOT gradient and obstruction. Mitral regurgitation may result from LVOTO or be a consequence of primary changes in the valve leaflets, potentially leading to the onset of dyspnea [25,26].

Genetic mutations lead to dysfunction in sarcomeric proteins, resulting in impaired interactions between actin and myosin and a disrupted switched-off state of the thin filament at low calcium concentrations [27,28]. This causes force generation to require a higher energy demand. The mutation-driven increase in myofilament calcium sensitivity leads to elevated cytosolic calcium buffering, directly affecting myocardial relaxation and increasing the risk of arrhythmogenesis. Additionally, there is a disruption of sarcolemmal functions and remodeling of the sarcoplasmic reticulum [29]. 

The expression of abnormal sarcomeric proteins due to genetic mutations in the atria and ventricles directly impacts electrical remodeling and contractile dysfunction, making HCM patients more susceptible to ventricular and supraventricular arrhythmias [30]. Additionally, diastolic dysfunction, mitral regurgitation, and increased intraventricular pressure can lead to tissue fibrosis in the left atrium, further increasing the likelihood of supraventricular arrhythmias. The most common arrhythmia among HCM patients is atrial fibrillation, with a prevalence between 19% and 30% [31,32,33]. Enlarged left atrial dimensions (>45 mm) are a strong predictor of atrial fibrillation in HCM patients [30,34,35].

Coronary pain, which is a direct consequence of myocardial ischemia, is one of the most common symptoms observed in patients with HCM [36]. Changes occurring in the vessels supplying the myocardium, such as luminal narrowing due to medial hypertrophy and intimal hyperplasia, lead to a reduction in the effective area of the microcirculation, resulting in a decrease in peak maximal vasodilator reserve [37]. Fibrosis, frequently seen in HCM patients, may further contribute to a reduction in the total cross-sectional area of the coronary microvasculature [38]. Additionally, myocardial hypertrophy and LVOTO exacerbate myocardial ischemia [23]. Consequently, myocardial ischemia and infarction may lead to the formation of left ventricular aneurysms, significantly increasing the risk of ventricular arrhythmias and HF [39].

## 3. Epidemiology and Risk Factors

HCM is a prevalent cardiac condition with an estimated prevalence of at least 1 in 500 individuals in the general population [40,41], as determined by echocardiography assessment of LVH [41]. Recent epidemiological studies indicate that the prevalence may be as high as 1 in 200 [42,43], attributable to the increased frequency of pathogenic mutations in sarcomere genes and the hereditary nature of the disorder [43]. Consequently, on a global scale, it is estimated that HCM may affect approximately 15 to 20 million individuals [36]. However, many individuals affected by HCM remain undiagnosed or are under-recognized for several reasons. These include the absence of overt symptoms and the presence of minor or subtle clinical and morphological manifestations. This issue is further exacerbated by a lack of clinical experience with HCM and limitations in diagnostic technology [42]. Despite advances in medical knowledge, gender- and race-related biases persist, resulting in delayed diagnoses and underutilization of specialized treatment for women [44,45] and minority populations [45,46].

The earliest reports of HCM were limited geographically to developed countries of North America and Western Europe. Currently, patients in at least 125 countries have been diagnosed with HCM [36,47]. Although the available data are incomplete, the clinical presentation of HCM, along with cardiac morphology and genetic underpinnings, appears to exhibit considerable consistency across various global regions [42].

**Figure 3:**
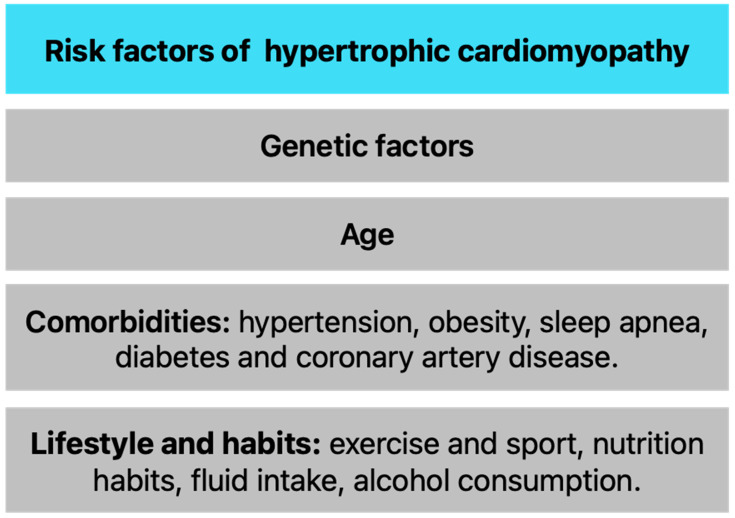
Risk factors of hypertrophic cardiomyopathy [40,42,48,49,50,51,52].

Several risk factors for HCM, as illustrated in Figure 3, are linked to its development and progression. These factors can be broadly classified into genetic predisposition, age, comorbidities, and lifestyle. Taking all risk factors into account allows us to understand the complexity of HCM.

Genetic factors are one of the most important factors in the pathogenesis of HCM, with mutations in sarcomeric protein genes being the most significant contributors [53]. Recognition of the hereditary nature of HCM aids in diagnosis and treatment and emphasizes the need for genetic counseling and family screening. Importantly, current studies show that the majority of patients with clinically diagnosed HCM (70%) do not actually carry sarcomeric gene mutations that are considered pathogenic [45]. Therefore, it is important to look for other risk factors.

Sex may be an important factor in predicting the course of HCM. Despite the autosomal dominant pattern of inheritance that would expect HCM to occur equally in both sexes, studies often indicate an overrepresentation of males, ranging from 55% to 70% [53,54]. Men are more likely diagnosed incidentally after identifying an abnormal electrocardiogram or heart murmur. Women are diagnosed at a later age than men; on average, HCM is diagnosed 6–13 years later [55]. Women are also more likely to have pathogenic sarcomeric variants than men (51% vs. 43%). Analysis from the Sarcomeric Human Cardiomyopathy Registry (SHaRe) showed that women were also more likely to have obstructive physiology (31.3% vs. 25.2%) and have a 50% higher mortality, a 50% higher risk of stroke, and a greater burden of prevalent and incident HF [54].

Age is another important risk factor in the context of HCM, influencing the onset of symptoms and the disease’s progression. The onset of HCM can occur at any age, but it is often diagnosed in adolescence or early adulthood. Symptoms such as chest pain, dyspnea, and syncope may first appear during periods of intense physical activity, especially in young athletes. It is recommended to exclude athletes with HCM from competitive high school and collegiate sports. Although HCM is not a generally progressive disease, HF symptoms may occur or increase in severity at any age, most frequently in mid-life due to LV outflow obstruction [45].

Among the co-occurring diseases that influence HCM, the following should be mentioned: hypertension, obesity, sleep apnea, diabetes, and coronary artery disease. Observational studies have shown that hypertension, obesity, and type 2 diabetes are more prevalent in individuals with HCM, but these could be secondary to reduced exercise. Hypertension is prevalent among individuals with HCM and can exacerbate LVH. Diastolic blood pressure (DBP) was identified as a key modifiable risk factor for sarcomere-negative HCM [40]. Obesity contributes to the overall cardiovascular risk and can complicate the management of HCM. Excess weight may worsen symptoms and increase the likelihood of HF [48]. Obstructive sleep apnoea is exceedingly prevalent in HCM [55] and it is known to increase the risk of factors that contribute to morbidity and mortality in HCM including arrhythmias, myocardial hypertrophy, and sudden cardiac death (SCD) [49]. Diabetes is associated with adverse cardiovascular outcomes and can worsen the prognosis for patients with HCM [50]. Patients with HCM may also develop coronary artery disease (CAD), which can complicate the clinical picture. The presence of ischemic heart disease can mask HCM symptoms and complicate treatment strategies. Patients with HCM who undergo coronary angiography, and those who have concomitant severe CAD, are at increased risk of death. This risk far exceeds historical death rates of CAD patients with normal left ventricular function [51].

However, lifestyle can also play a role in HCM progression. Strenuous physical exertion, particularly in competitive sports, can exacerbate symptoms in individuals with HCM. Intense exercise may trigger arrhythmias or lead to syncope, making it crucial for individuals diagnosed with HCM to undergo thorough assessments before participating in strenuous activities. Conversely, recreational exercise should be recommended for most people with HCM. A sedentary lifestyle can contribute to obesity and other cardiovascular risk factors, which may worsen the overall condition of patients with HCM. Patients should avoid large meals and reduce levels of postprandial activity. Proper fluid intake is also important, dehydration can cause deterioration of the left ventricular contraction dynamics. Finally, patients with HCM should consume alcohol in moderation, as it has been shown to decrease arterial blood pressure and increase systolic anterior motion severity and degree of intraventricular obstruction [52].

It is important to mention the risk factors for SCD in HCM. Such factors include a family history of SCD, unexplained syncope, non-sustained ventricular tachycardia (NSVT), maximal left ventricular wall thickness, abnormal exercise blood pressure response, and left ventricular systolic dysfunction. However, it is difficult to predict SCD with absolute certainty, as the annual incidence of SCD in adult patients with HCM is approximately 1% [56].

## 4. Diagnosis and Treatment 

While HCM, a primary form of cardiac hypertrophy, has been in existence for a long period, its diagnosis has improved significantly in the past few decades from simple echocardiographic evaluations to a more complex, multimodal approach embracing advanced imaging, and genetic and biomarker studies. This development gives credit to the increased recognition of HCM as a complex clinical condition as well as highlighting the need for adequate definition to facilitate appropriate diagnosis and management [13].

According to current criteria, HCM diagnosis is based on LV thickness. In most age groups, a maximum LV thickness of ≥15 mm at any site in the chamber is consistent with the identification of HCM, or a maximum LV wall thickness of ≥13 mm for patients with a first-degree relative with confirmed HCM. Additionally, 13 to 14 mm can be diagnostic, particularly when associated with HCM family history, typical dynamic outflow obstruction, or distinctly abnormal electrocardiography (ECG) patterns [36]. Clinical evaluation is equally critical, with emphasis on the presence of symptoms like dyspnea, chest pain, syncope, and a family history of HCM or SCD [57]. However, these methods are limited since they are predominantly reliant on two-dimensional (2D) echocardiography by several factors, including operator dependency, variability in image quality, and the inability to detect subtle or localized hypertrophic changes, which could lead to underdiagnosis or misclassification of the disease [58].

Furthermore, the ESC guidelines highlight the significance of risk stratification for SCD, incorporating factors such as family history of SCD, unexplained syncope, extreme LV hypertrophy (≥30 mm), and the presence of LGE on CMR [59]. This comprehensive approach ensures that patients at higher risk receive appropriate interventions, such as implantable cardioverter-defibrillators (ICDs), to prevent adverse outcomes [60].

### 4.1. Advancements in Imaging Techniques

The advent of three-dimensional (3D) echocardiography marked a significant advancement in diagnostic capabilities for HCM. Unlike 2D echocardiography, 3D echocardiography offers enhanced spatial resolution and more accurate quantification of myocardial thickness and LV geometry, addressing previous limitations related to spatial resolution and anatomical variability [61]. This modality allows for a comprehensive assessment of the entire myocardium, enabling the detection of localized hypertrophic regions that might be overlooked in 2D imaging [62].

Cardiovascular magnetic resonance (CMR) imaging has emerged as a cornerstone in the contemporary diagnostic framework for HCM. CMR provides high-contrast, high-resolution images that facilitate precise measurements of myocardial thickness, identification of myocardial fibrosis through late gadolinium enhancement (LGE), and detailed evaluation of LVOTO [63]. The ability of CMR to characterize tissue properties makes it invaluable for differentiating HCM from other forms of cardiac hypertrophy, such as amyloidosis or athlete’s heart, by identifying specific patterns of fibrosis and hypertrophy as presented in Table 1 [64].

Speckle-tracking echocardiography (STE) has further refined the diagnostic process by enabling the assessment of the myocardial strain and detecting subclinical myocardial dysfunction [65]. STE analyzes the deformation of myocardial fibers during the cardiac cycle, providing insights into myocardial mechanics that are not apparent through conventional echocardiographic techniques. This capability is particularly useful in identifying early hypertrophic changes and in patients with borderline hypertrophy where traditional methods may be inconclusive [66].

### 4.2. Genetic Testing

The integration of genetic testing into the diagnostic algorithm for HCM represents a significant paradigm shift. HCM is often inherited in an autosomal dominant pattern, with mutations in genes encoding sarcomeric proteins, such as MYH7 and MYBPC3, being the most common genetic determinants [67]. Genetic testing not only confirms the diagnosis in individuals with a positive family history but also facilitates cascade screening of at-risk relatives, thereby enabling early detection and management [68]. Moreover, genotype–phenotype correlations have provided deeper insights into the variability of clinical presentations and disease progression, allowing for more personalized risk stratification and therapeutic interventions [69].

Advancements in next-generation sequencing (NGS) have enhanced the efficiency and comprehensiveness of genetic testing, enabling the simultaneous analysis of multiple genes associated with HCM [70]. This approach has increased the diagnostic yield and has identified novel genetic variants that contribute to the heterogeneous nature of HCM [71]. Furthermore, the identification of pathogenic variants has implications for prognosis, as certain mutations are associated with a higher risk of adverse outcomes, including SCD [72].

### 4.3. Biomarkers

The use of biomarkers has become a more sensitive approach in enabling a diagnosis of HCM. The elevated levels of N-terminal pro-brain natriuretic peptide (NT-proBNP) and high-sensitivity cardiac troponin I (hsTnI) are relevant in the HCM because they quantify myocardial stress and damage [73]. They help to discriminate HCM from other etiologies leading to LVH like hypertensive heart disease or athlete’s heart by providing objective evidence of myocardial impairment [74]. Moreover, other biomarkers such as galectin-3 and growth differentiation factor (GDF-15) are investigated to determine their role as fibrosis and inflammation biomarkers for diagnosis, respectively [75].

### 4.4. Artificial Intelligence and Machine Learning

Using artificial intelligence (AI) and machine learning (ML) technologies is a novel step forward in the process of HCM diagnosis. These technologies also help to enhance the interpretation of imaging and genomic data by harnessing complex and deeper predictive analytics capabilities that are beyond what typical analytics can offer [76]. The tools that are automated by AI are capable of handling enormous amounts of data in a bid to enhance the level of accuracy in diagnosis, and assess the likelihood of disease progression and danger to the lives of the patients presenting SCD [77]. For example, studies have shown that traces of diabetes in patients can be detected using machine learning algorithms in echocardiographic images and magnetic resonance imaging that may raise suspicion of undiagnosed HCM [78]. Moreover, genetic data interpretation software, such as DeepVariant (v1.6.1) and Clairvoyante (v3), equipped with machine learning tools aids in quick and accurate filtering of the disease-causing genetic variants as well as their interpretation in the clinical context [79].

### 4.5. Multimodal Diagnostic Approach

Most commonly, the latest diagnostic tools used in HCM or heart muscle disease are based on multimodality imaging, genetic testing, and biomarker assessment for effective and complete diagnosis [80]. Hence, this is particularly advantageous for such patients whose echocardiographic assessments are not very clear in pictures or whose families have a strong history of HCM but may not meet other requirements [81]. Clinicians can determine the nature and extent of myocardial hypertrophy and associated risks for its complications, and choose strategies for the management of the patients that meet their needs [82].

In one scenario, a patient suspected to have HCM may be subjected to a 3D echocardiogram in anticipation of measuring the thickness and geometry of the walls of the LV which will be followed by a CMR test to check for LVOTO-associated myocardial fibrosis. At the same time, blood may be drawn to test for the presence of genes responsible for the condition. Enzymes that suppress the stress response of the heart can also be detected. Such an elaborate and thorough work up is ideal in ensuring that no stone is left unturned in the process of diagnosing the disease and which helps in its early treatment [83].

### 4.6. Differential Diagnosis and Advanced Imaging Techniques

The treatment is dependent on proper differentiation of the HCM with other reasons for LV hypertrophy. Patients with hypertensive heart or Fabry disease, younger athletes with athlete’s heart, and older people with cardiac amyloidosis may show the same echocardiography pictures but need different treatment [82]. Among such advanced diagnostic methods, structural heart imaging can be mentioned, especially using CMR. For example, CMR LGE is mainly observed in patients with HCM and patients with a high risk of arrhythmia, whereas patients with CAD have a more pronounced pattern of diffuse interstitial fibrosis [84].

Cardiac scintigraphic methods are gradually beginning to occupy the niche of imaging and quantifying metabolic and fibrotic activity in hypertrophied myocardium [85]. Positron emission tomography (PET) can assess glucose metabolism through myocardial tracer uptake and inflammation through tracer enhancement in areas of active inflammation, thereby adding functional PET imaging as a soft imaging complement to hard structural imaging [86]. This technique has been effective in the differentiation of HCM and infiltrative cardiomyopathies, and in the assessment of myocardial metabolism, which bears both diagnostic and clinical therapy targeting potentials [87].

### 4.7. Contemporary Treatment Strategies

Current treatment for HCM primarily relies on pharmacological interventions. The objectives of pharmacotherapy are to alleviate symptoms, enhance the quality of life, and prevent sudden cardiac death [88,89]. Beta-blockers that do not cause vasodilation are among the most utilized and effective treatment options, as they reduce LVOTO and prolong the diastolic phase through their negative inotropic and chronotropic effects [89]. These medications are particularly effective in cases of latent obstruction triggered by physical exertion and can also positively influence gradients at rest [89,90]. 

In a randomized controlled trial involving patients with obstructive HCM treated with either metoprolol or a placebo for two weeks, those receiving metoprolol demonstrated a significant reduction in the LVOT gradient at rest (25 mmHg compared to 72 mmHg) and during exertion (28 mmHg compared to 62 mmHg). Furthermore, these patients exhibited an improvement in their functional class according to the NYHA (New York Heart Association) scale compared to the placebo group [91]. For patients with beta-blocker intolerance or inadequate efficacy, calcium channel antagonists, such as verapamil, may serve as alternative treatment options, as they also facilitate improved ventricular filling and reduction in outflow obstruction [92]. If symptoms persist despite the use of these pharmacological agents, the addition of disopyramide—a Class Ia antiarrhythmic agent with significant negative inotropic effects—may be considered [92,93]. Although this therapeutic strategy has been shown to alleviate symptoms and enhance quality of life, it has not yet demonstrated the ability to modify the natural progression of the disease or improve mortality rates [88,89,90,94]. 

Despite the implementation of optimal medical therapy, patients who do not respond to conservative treatment may benefit from septal reduction therapy, which leads to symptom relief in 90–95% of cases. Treatment options include septal myectomy and percutaneous alcohol septal ablation [95,96]. According to the guidelines from the ACC and the ESC regarding the management of HCM, surgical myectomy is considered the gold standard for septal reduction. This procedure involves the resection of a small portion of muscle from the interventricular septum to decrease the left ventricular outflow tract gradient [57,95,97]. Conversely, alcohol septal ablation has emerged as an alternative to myectomy for selected patients, particularly those who are elderly or have comorbidities [88,98]. 

In addition to the aforementioned treatment methods for HCM, patient education is a crucial component of therapy. Recommended interventions for patients include maintaining proper hydration levels and achieving a healthy body weight. Current guidelines from the ACC Foundation and the AHA advocate for low-intensity aerobic exercise to improve and sustain cardiovascular fitness. Patients are also advised to avoid excessive alcohol consumption, stimulants, and exposure to extreme temperatures, which can trigger abrupt changes in cardiac preload [88,90].

### 4.8. Challenges in Diagnosing and Managing HCM

HCM remains among the most challenging cardiovascular conditions to diagnose and manage, given its genetic heterogeneity, variable presentation, and substantial associated risks, including SCD and progression to HF [99]. HCM symptoms range widely, from asymptomatic cases to progressive dyspnea, angina, and syncope, further complicating early detection and risk assessment [100].

Diagnostic approaches—primarily echocardiography [101] and cardiac MRI [102]—are essential for delineating LVH and detecting LVOTO [80]. However, these studies require specialized interpretation to differentiate HCM from other causes of LVH, such as athlete’s heart and hypertensive heart disease. Nevertheless, they pose advantages as compared to the traditional techniques. CMR further elevates diagnostic capabilities by offering detailed insights into myocardial tissue characteristics, including the detection of myocardial fibrosis, which is a hallmark of HCM. CMR is instrumental in evaluating LVOTO and identifying areas of late gadolinium enhancement, which correlate with adverse outcomes. The integration of these advanced imaging modalities with traditional ECG enhances risk stratification, enabling clinicians to better assess arrhythmic risk and guide management strategies. The incorporation of machine learning techniques for classifying QRS complexes, as discussed in the studies by De Marco et al. [103,104], offers promising advancements in detecting premature ventricular contractions (PVCs) and could enhance the diagnostic capabilities for HCM. By analyzing QRS morphology through automated algorithms, machine learning can improve the accuracy of arrhythmia detection and assist in differentiating HCM-related abnormalities from other cardiac conditions. This approach, when integrated with advanced imaging modalities like CMR and 3D echocardiography, can further refine risk stratification and management strategies for HCM patients [105].

While genetic testing can help identify pathogenic mutations, it introduces added complexity due to high costs, incomplete penetrance, and significant variability in clinical expression, complicating risk stratification efforts [106].

Risk stratification, especially regarding SCD, remains a major challenge, as current criteria for ICD implantation—such as family history, wall thickness, and presence of ventricular arrhythmias—do not fully capture all risk factors, potentially leaving some patients at increased or ambiguous risk [107]. In response, recent therapeutic advancements show promise in reshaping HCM management, particularly with targeted therapies like Mavacamten. Therefore, this review examines the clinical challenges in HCM management and explores Mavacamten’s pharmacologic mechanism, clinical efficacy, and its potential to redefine the future landscape of HCM treatment.

### 4.9. Future Directions and Emerging Technologies

Future development of the diagnostic environment of HCM remains optimistic due to the progress in the investigation of novel imaging techniques and molecular medicine devices. Myocardial tissue characteristics and function have advanced to be critiqued in more detail using techniques like diffusion tensor imaging (DTI) and molecular imaging [108]. Furthermore, the recent development of wearable devices and remote monitoring systems may also be beneficial for the long-term monitoring of cardiac function in HCM patients through remote healing, which will avert later lesions of the disease and timely intervention [109].

Further examination into the genetic basis of HCM also considers glass clerestory, in which current research seeks to unravel the range of genetic variations responsible for the disease as well as its functionalities [110]. This understanding will not only improve the accuracy of genetic tests but also help in finding treatment options that target the root causes of HCM at the molecular level [111].

In addition, the principles of the individualization of therapy based on genetic markers, biomarkers, and imaging, as well as HCM itself, will change for the better. Effective measures for the prevention and treatment of patients will also be implemented due to the fact that every scheme will be the most optimal for this specific clinical example [112].

**Table 1:** Summarizing imaging modalities for diagnosing HCM, incorporating traditional and advanced technologies with its strengths and limitations. Each modality offers unique diagnostic strengths, enhancing detection accuracy, and stratifying risk factors of HCM patients.

Modality	Diagnostic Role in HCM	Strengths	Limitations
3D Echocardiography	Measures LV wall thickness and geometry with high precision; essential for initial assessment of LV hypertrophy [61,62].	Real-time imaging, optimal for initial hypertrophy assessment.	Limited spatial resolution, less effective for fibrosis detection.
Cardiac MRI (CMR)	Superior for detecting myocardial fibrosis and assessing LVOTO; CMR LGE patterns assist in stratifying risk of arrhythmia and SCD.	High spatial resolution, valuable for high-risk assessment with fibrosis quantification [64].	Costly, requires specialized interpretation, limited access.
Positron Emission Tomography (PET)	Assesses metabolic and inflammatory activity in myocardium; differentiates HCM from infiltrative cardiomyopathies, such as amyloidosis.	Useful for assessing fibrosis extent and glucose metabolism in myocardium [86,87].	High cost, limited availability, radiation exposure.
AI-Enhanced Echocardiography	Supports enhanced data analysis, identifies undiagnosed conditions (e.g., diabetes as a co-factor in HCM).	Increased diagnostic accuracy and disease progression prediction through large-scale data processing [78,79].	Requires significant computational resources, ethical considerations.
Multimodal Approach	Combines echocardiography, CMR, genetic, and biomarker assessment to provide a comprehensive diagnosis.	Comprehensive and tailored diagnostic profile, crucial in ambiguous cases with strong family history [82,83].	High cost, complex integration of data.

## 5. Mavacamten—Characteristics and Scientific Studies

Mavacamten, or MYK-461 chemically known as 6-[[(1S)-1-phenylethyl]amino]-3-propan-2-yl-1H-pyrimidine-2,4-dione, belongs to the pyrimidine group of drugs [113,114]. It is a reversible, selective, and allosteric small-molecule inhibitor of cardiac myosin [115,116,117]. The drug was approved by the U.S. Food and Drug Administration in April 2022 and it trade name is Camzyos™ [117,118]. The drug was developed and created by MyoKardia, and later its production was taken over by Bristol-Myers Squibb Pharma EEIG.

This drug comes in capsules in the following dosages: 2.5 mg, 5 mg, 10 mg, and 15 mg [118]. The recommended dose of the drug is initially 5 mg once daily regardless of meals. This dose is then modified and is determined by the LVOT gradient [119]. Dosage titration can be left at an average of every 4 weeks, reaching a maximum of 15 mg per day so that the plasma concentration is between 350 and 700 ng/mL [120].

The drug is metabolized by more than 70% by CY2C19, with smaller percentages by CYP3A4 and CYP2C9. The drug is more than 80% excreted in the urine and less than 10% in the feces. The pharmacokinetics of the drug are not affected by race, ethnicity, age, or gender [118]. Bioavailability after oral administration is more than 85% reaching maximum concentration 1 h after intake. The drug takes quite a long time to be eliminated from the body, having a terminal half-life of about 8 days on average and a large volume of distribution [113,118]. This period of about 8 days applies to those with CYP2C19 enzymes that metabolize properly. Those who metabolize poorly may have an extended half-life of up to 23 days [118]. Mavacamten’s mechanism is based on the fact that it is an inhibitor of cardiac myosin ATPase [121,122]. To understand the action of this drug, it is necessary to start with the fact that myosin is a dimeric, enzymatic, protein that builds the sarcomeres of the heart. Myosin contains an enzyme, adenosine triphosphate (ATP)ase, which breaks down ATP to allow the interaction between actin and myosin, resulting in shortened sarcomeres. Patients with HCM have a pathological increase in the amount of interaction between actin and myosin, and as a result, these patients have poor relaxation, overly dynamic contraction, and increased energy use [123,124]. Thanks to mavacamten, there is a reduction in the number of myosin heads available to bind to actin which results in a reduction in the number of actin-myosin cross-bridges in both systole and diastole. By reducing the number of bridges, LVOT pressure is normalized and cardiac cavities are filled, which has a positive effect on the patient’s performance and reduces symptoms such as chest pain and dyspnea [114]. 

Thus, this drug decreases the affinity of actin to myosin, which results in a decrease in myocardial contractility and a decrease in the force generated by sarcomeres, so that ventricular hypertrophy, microfilament breakdown, and fibrosis of myocardial tissue in the heart do not occur [113,123,124].

It is worth noting that the mechanism of action of mavacamten is significantly different from the large and well-known group of drugs used in patients with HCM, namely beta-blockers or non-dihydropyridine calcium channel blockers (CCBs) [125]. 

The mechanism of beta-blocker drugs, which are often used as first-line therapy, is based on sympathetic modulation, resulting in a decrease in ventricular contractility and heart rate, resulting in prolonged ventricular relaxation and going further to increase LVOT patency [91]. 

Calcium channel blockers, most commonly used in patients with non-obstructive HCM, work by improving earlier ventricular diastole and prolonging left ventricular filling time [126]. Mavacamten, on the other hand, works by targeting excessively contracted cardiac fibers, which improves not only LVOT patency but also fitness and reduces left ventricular and atrial hypertrophy. An important difference is that it is the only and first drug whose use is completely focused on the treatment of a single disease, namely HCM [127].

The drug is used to increase the left ventricular end-diastolic volume and, most importantly, save the patient from the need for surgery [128,129].

Mavacamten is approved in adults with symptomatic NYHA II-III HCM HF and is the first to be directly approved in patients with obstructive HCM pathology [117,119,120].

We use this drug in patients with oHCM who have symptoms such as dyspnea, syncope, and chest pain despite treatment with beta-blockers and calcium channel blockers [115].

It is worth noting that treatment should not be started if the left ventricle ejection fraction (LVEF) is below 55% because systolic function may deteriorate [120]. 

Patients are advised to undergo echocardiography at 4, 8, and 12 weeks after starting treatment, and every 12 weeks thereafter [115].

It is worth noting that the use of this drug in patients from special groups. Patients with mild to moderate hepatic impairment have significantly increased exposure to the drug, while the effect is unknown in those with severe liver disease, so the physician must be particularly cautious. Animal studies have shown the toxic effects of the drug on the fetus of the mother using the drug, so contraception is recommended during drug use and 4 months after discontinuation. The effect on the child during lactation remains unknown for the time being. The same applies to the pediatric population [120].

**Figure 4:**
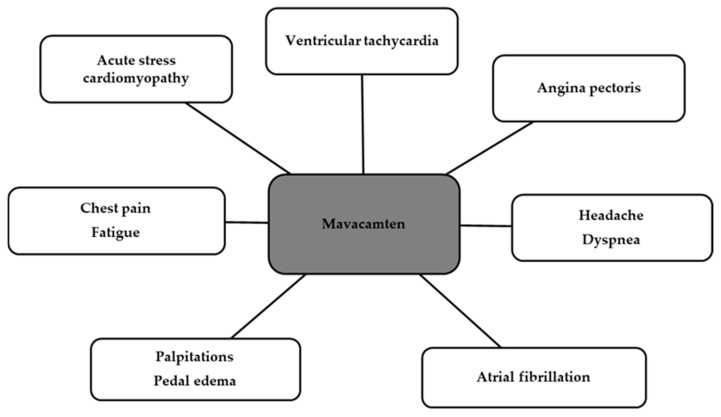
Possible adverse reactions occurring during Mavacamten use [120].

As for the safety of the cardiac myosin inhibitor, the most common adverse effects were atrial fibrillation and decreased left ventricular ejection fraction, but these were not permanent [128]. Figure 4 shows other side effects occurring in patients [120].

In the situation of ingesting too large a dose of the drug, systolic dysfunction may occur, and the patient will experience an exacerbation of HF. It has been shown that activated charcoal can reduce the absorption of the drug into the bloodstream if given within 2 h of ingestion [120].

Mavacamten has been the subject of extensive research to evaluate its efficacy and safety for the treatment of HCM, particularly its obstructive form (oHCM). Among the critical clinical studies on Mavacamten, the EXPLORER-HCM trial is one of the most significant [104]. EXPLORER-HCM was a double-blind, randomized, placebo-controlled phase 3 trial that included 251 symptomatic patients with obstructive HCM, characterized by NYHA class II or III HF symptoms and an LVOT gradient of ≥50 mmHg at rest or with provocation. The primary outcome was a composite endpoint of a ≥1.5 mL/kg/min improvement in peak oxygen consumption (peak VO2), reflecting enhanced exercise capacity, coupled with a reduction in NYHA class by at least one level [130]. The findings were highly encouraging. Thirty-seven percent of patients receiving Mavacamten reached the primary endpoint compared with seventeen percent in the placebo group. Mavacamten also led to a significant reduction in the LVOT gradient, with an average reduction of 36 mmHg from baseline, compared with a 16 mmHg reduction in the placebo group. Additionally, secondary measures, such as the Kansas City Cardiomyopathy Questionnaire (KCCQ), demonstrated marked improvements in patient-reported quality of life and physical function [130,131,132]. Key outcomes from this trial are shown in Table 2 below [130].

These results not only highlighted the clinical significance of Mavacamten but also underscored its potential to address both symptomatic relief and the structural pathophysiology of HCM. By directly targeting the myosin-heavy chain and reducing hypercontractility, Mavacamten showed promise in reshaping how obstructive HCM could be managed in the future [133]. Following the success of EXPLORER-HCM, the VALOR-HCM trial was conducted to investigate Mavacamten’s role in reducing the need for invasive procedures like septal myectomy or alcohol septal ablation in patients with severe obstructive HCM [134]. This phase 3 trial enrolled patients who were either scheduled for or eligible for septal reduction therapy (SRT) and randomized them to receive Mavacamten or placebo for 16 weeks. The primary endpoint was the percentage of patients who continued to meet the criteria for SRT at the end of the trial [134,135]. The outcomes of VALOR-HCM were striking. By week 16, only 18% of patients in the Mavacamten group still met the criteria for SRT, compared to 77% in the placebo group, demonstrating the drug’s ability to significantly reduce the need for these invasive procedures [135,136]. The trial also reported notable improvements in LVOT gradient reduction, exercise capacity, and HF symptoms. These results are significant not only because they show a reduction in SRT but also because they signal the potential for Mavacamten to change the standard approach to managing severe HCM cases. While septal reduction therapies have been effective for some patients, they carry risks associated with invasive procedures and extended recovery periods. By offering a pharmacological alternative, Mavacamten has the potential to simplify and improve long-term outcomes for many patients [137,138]. In addition to its role in treating obstructive HCM, Mavacamten has been evaluated in patients with nHCM through the MAVERICK-HCM trial, a phase 2 study that enrolled 69 symptomatic patients with nHCM. These patients typically suffer from impaired diastolic function and HF symptoms despite the absence of significant LVOTO [139]. The primary focus of the MAVERICK-HCM trial was to assess the safety and tolerability of Mavacamten and to evaluate biomarkers of myocardial stress, such as NT-proBNP and cardiac troponin. The results from MAVERICK-HCM indicated that Mavacamten was generally well-tolerated, with a favorable safety profile [140,141]. Patients receiving Mavacamten showed reductions in NT-proBNP and cardiac troponin levels, signaling a decrease in myocardial stress. Although the study was not designed to evaluate major clinical outcomes, the reductions in biomarkers were promising, suggesting that Mavacamten may also benefit patients with non-obstructive HCM [139,141]. However, further research is needed to confirm these preliminary findings and to determine the drug’s clinical utility in this subgroup [141]. As Mavacamten’s clinical applications expand, its safety and efficacy continue to be the subject of ongoing studies. The MAVA-LTE trial is a study that follows patients who participated in previous Mavacamten trials to assess the durability of the drug’s effects and its safety over longer periods. Initial data from this trial indicate that Mavacamten’s benefits in reducing LVOT gradients and improving symptoms are sustained over time, without significant adverse effects. This durability is crucial, as HCM is a chronic condition that often requires long-term management, and patients need therapies that offer sustained benefits without compromising safety [125,131]. Compared to standard invasive treatments for obstructive HCM, such as septal myectomy or alcohol septal ablation, Mavacamten offers several potential advantages. Traditional interventions aim to relieve LVOTO by surgically reducing septal thickness or creating a localized septal infarct, procedures that can effectively alleviate symptoms but carry risks associated with invasive surgery, including extended recovery time, potential complications, and, in some cases, the need for re-intervention [130,135,142]. In contrast, Mavacamten targets the underlying hypercontractility of the myocardium pharmacologically, reducing LVOT gradient and symptoms without the invasiveness of surgery [115,143]. The EXPLORER-HCM trial demonstrated that Mavacamten could significantly improve functional capacity and symptoms, offering an alternative to septal reduction therapies for suitable patients [115,130,135]. In the VALOR-HCM trial, for instance, Mavacamten reduced the percentage of patients eligible for SRT by 77% compared to placebo, indicating that for many patients, pharmacological management could serve as a viable alternative to septal reduction therapies [135,142,144]. This reduction in the need for invasive procedures suggests that Mavacamten could be particularly valuable for patients with contraindications to surgery or those who wish to avoid procedural risks [130,135,142]. Recent comparisons in the literature, such as a study by Scholtz et al., have highlighted the advantages of Mavacamten over traditional alcohol septal ablation, noting its favorable safety profile and sustained efficacy [142]. Furthermore, Tuohy et al. have emphasized the future direction of treatment for hypertrophic cardiomyopathy, suggesting that Mavacamten may be pivotal in shifting treatment paradigms towards less invasive options [145]. Taken together, outcomes from EXPLORER-HCM and VALOR-HCM suggest that Mavacamten may reshape the standard approach to obstructive HCM treatment, positioning it as a promising pharmacological alternative alongside traditional interventions [115,130,135,143,145].

## 6. Conclusions

HCM is a complex and heterogeneous condition that requires a thorough approach to diagnosis and management. Recent advancements in diagnostic techniques, particularly the integration of three-dimensional echocardiography, cardiovascular magnetic resonance imaging, and genetic testing, have greatly improved our ability to identify and stratify patients who are at risk for adverse clinical outcomes. This shift towards a multimodal diagnostic strategy not only enhances the accuracy of HCM detection but also facilitates individualized therapeutic interventions tailored to the specific characteristics of each patient’s condition.

The emergence of Mavacamten marks a significant milestone in the management of obstructive HCM, underscoring the potential of targeted pharmacological treatments to alleviate symptoms and reduce the reliance on invasive procedures. Clinical trials, particularly EXPLORER-HCM and VALOR-HCM, have shown encouraging results, demonstrating that Mavacamten can markedly improve exercise capacity and quality of life while normalizing left ventricular outflow tract pressures. As research in this area advances, further studies will be essential to assess the long-term safety and efficacy of Mavacamten, especially concerning non-obstructive variants of HCM.

## Figures and Tables

**Table 2:** Key Outcomes from the EXPLORER-HCM Trial [130].

Outcome Measure	Mavacamten Group (n = 123)	Placebo Group (n = 128)
Primary Endpoint Achievement	37%	17%
NYHA Class Improvement (≥1 Class)	65%	31%
Peak post-exercise reduction in LVOT Gradient (mmHg)	48 mmHg	11 mmHg
At least a 3 mL/kg/min increase in pVO_2_ and + at least one class improvement in NYHA class	20%	8%
KCCQ Quality of Life Score	+9 points more than in placebo	-

NYHA, New York Heart Association; LVOT, Left Ventricular Outflow Tract; mmHg, millimeters of mercury; KCCQ, Kansas City Cardiomyopathy Questionnaire; pVO_2,_ peak oxygen consumption.

## References

[1] Maron B.J., Towbin J.A., Thiene G., Antzelevitch C., Corrado D., Arnett D., Moss A.J., Seidman C.E., Young J.B., American Heart A. (2006). Contemporary definitions and classification of the cardiomyopathies: An American Heart Association Scientific Statement from the Council on Clinical Cardiology, Heart Failure and Transplantation Committee; Quality of Care and Outcomes Research and Functional Genomics and Translational Biology Interdisciplinary Working Groups; and Council on Epidemiology and Prevention. Circulation.

[2] McKenna W.J., Maron B.J., Thiene G. (2017). Classification, Epidemiology, and Global Burden of Cardiomyopathies. Circ. Res..

[3] Elliott P., Andersson B., Arbustini E., Bilinska Z., Cecchi F., Charron P., Dubourg O., Kühl U., Maisch B., McKenna W.J. (2008). Classification of the cardiomyopathies: A position statement from the European Society of Cardiology Working Group on Myocardial and Pericardial Diseases. Eur. Heart J..

[4] Arbustini E., Narula N., Tavazzi L., Serio A., Grasso M., Favalli V., Bellazzi R., Tajik J.A., Bonow R.O., Fuster V. (2014). The MOGE(S) classification of cardiomyopathy for clinicians. J. Am. Coll. Cardiol..

[5] Geske J.B., Ommen S.R., Gersh B.J. (2018). Hypertrophic Cardiomyopathy: Clinical Update. JACC Heart Fail..

[6] Mazur M., Braksator W., Popjes E. (2024). Hypertrophic Cardiomyopathy: From Medical Treatment to Advanced Heart Failure Therapies. Curr. Cardiol. Rep..

[7] Maron B.J., Maron M.S., Semsarian C. (2012). Genetics of hypertrophic cardiomyopathy after 20 years: Clinical perspectives. J. Am. Coll. Cardiol..

[8] Ho C.Y., Charron P., Richard P., Girolami F., Van Spaendonck-Zwarts K.Y., Pinto Y. (2015). Genetic advances in sarcomeric cardiomyopathies: State of the art. Cardiovasc. Res..

[9] Thierfelder L., Watkins H., MacRae C., Lamas R., McKenna W., Vosberg H.P., Seidman J.G., Seidman C.E. (1994). Alpha-tropomyosin and cardiac troponin T mutations cause familial hypertrophic cardiomyopathy: A disease of the sarcomere. Cell.

[10] Van Driest S.L., Ellsworth E.G., Ommen S.R., Tajik A.J., Gersh B.J., Ackerman M.J. (2003). Prevalence and spectrum of thin filament mutations in an outpatient referral population with hypertrophic cardiomyopathy. Circulation.

[11] Coppini R., Ho C.Y., Ashley E., Day S., Ferrantini C., Girolami F., Tomberli B., Bardi S., Torricelli F., Cecchi F. (2014). Clinical phenotype and outcome of hypertrophic cardiomyopathy associated with thin-filament gene mutations. J. Am. Coll. Cardiol..

[12] Van Driest S.L., Vasile V.C., Ommen S.R., Will M.L., Tajik A.J., Gersh B.J., Ackerman M.J. (2004). Myosin binding protein C mutations and compound heterozygosity in hypertrophic cardiomyopathy. J. Am. Coll. Cardiol..

[13] Marian A.J., Braunwald E. (2017). Hypertrophic Cardiomyopathy: Genetics, Pathogenesis, Clinical Manifestations, Diagnosis, and Therapy. Circ. Res..

[14] Witjas-Paalberends E.R., Piroddi N., Stam K., van Dijk S.J., Oliviera V.S., Ferrara C., Scellini B., Hazebroek M., ten Cate F.J., van Slegtenhorst M. (2013). Mutations in MYH7 reduce the force generating capacity of sarcomeres in human familial hypertrophic cardiomyopathy. Cardiovasc. Res..

[15] Witjas-Paalberends E.R., Güçlü A., Germans T., Knaapen P., Harms H.J., Vermeer A.M., Christiaans I., Wilde A.A., Dos Remedios C., Lammertsma A.A. (2014). Gene-specific increase in the energetic cost of contraction in hypertrophic cardiomyopathy caused by thick filament mutations. Cardiovasc. Res..

[16] Crilley J.G., Boehm E.A., Blair E., Rajagopalan B., Blamire A.M., Styles P., McKenna W.J., Ostman-Smith I., Clarke K., Watkins H. (2003). Hypertrophic cardiomyopathy due to sarcomeric gene mutations is characterized by impaired energy metabolism irrespective of the degree of hypertrophy. J. Am. Coll. Cardiol..

[17] Sedaghat-Hamedani F., Kayvanpour E., Tugrul O.F., Lai A., Amr A., Haas J., Proctor T., Ehlermann P., Jensen K., Katus H.A. (2018). Clinical outcomes associated with sarcomere mutations in hypertrophic cardiomyopathy: A meta-analysis on 7675 individuals. Clin. Res. Cardiol..

[18] Li R.K., Li G., Mickle D.A., Weisel R.D., Merante F., Luss H., Rao V., Christakis G.T., Williams W.G. (1997). Overexpression of transforming growth factor-beta1 and insulin-like growth factor-I in patients with idiopathic hypertrophic cardiomyopathy. Circulation.

[19] Lan F., Lee A.S., Liang P., Sanchez-Freire V., Nguyen P.K., Wang L., Han L., Yen M., Wang Y., Sun N. (2013). Abnormal calcium handling properties underlie familial hypertrophic cardiomyopathy pathology in patient-specific induced pluripotent stem cells. Cell Stem Cell.

[20] Marian A.J. (2000). Pathogenesis of diverse clinical and pathological phenotypes in hypertrophic cardiomyopathy. Lancet.

[21] Jan M.F., Todaro M.C., Oreto L., Tajik A.J. (2016). Apical hypertrophic cardiomyopathy: Present status. Int. J. Cardiol..

[22] Jan M.F., Tajik A.J. (2017). Modern Imaging Techniques in Cardiomyopathies. Circ. Res..

[23] Patel P., Dhillon A., Popovic Z.B., Smedira N.G., Rizzo J., Thamilarasan M., Agler D., Lytle B.W., Lever H.M., Desai M.Y. (2015). Left Ventricular Outflow Tract Obstruction in Hypertrophic Cardiomyopathy Patients Without Severe Septal Hypertrophy: Implications of Mitral Valve and Papillary Muscle Abnormalities Assessed Using Cardiac Magnetic Resonance and Echocardiography. Circ. Cardiovasc. Imaging.

[24] Maron M.S., Olivotto I., Zenovich A.G., Link M.S., Pandian N.G., Kuvin J.T., Nistri S., Cecchi F., Udelson J.E., Maron B.J. (2006). Hypertrophic cardiomyopathy is predominantly a disease of left ventricular outflow tract obstruction. Circulation.

[25] Maron M.S., Olivotto I., Betocchi S., Casey S.A., Lesser J.R., Losi M.A., Cecchi F., Maron B.J. (2003). Effect of left ventricular outflow tract obstruction on clinical outcome in hypertrophic cardiomyopathy. N. Engl. J. Med..

[26] Hang D., Schaff H.V., Nishimura R.A., Lahr B.D., Abel M.D., Dearani J.A., Ommen S.R. (2019). Accuracy of Jet Direction on Doppler Echocardiography in Identifying the Etiology of Mitral Regurgitation in Obstructive Hypertrophic Cardiomyopathy. J. Am. Soc. Echocardiogr..

[27] Spudich J.A. (2019). Three perspectives on the molecular basis of hypercontractility caused by hypertrophic cardiomyopathy mutations. Pflug. Arch..

[28] Tardiff J.C., Carrier L., Bers D.M., Poggesi C., Ferrantini C., Coppini R., Maier L.S., Ashrafian H., Huke S., van der Velden J. (2015). Targets for therapy in sarcomeric cardiomyopathies. Cardiovasc. Res..

[29] Coppini R., Ferrantini C., Yao L., Fan P., Del Lungo M., Stillitano F., Sartiani L., Tosi B., Suffredini S., Tesi C. (2013). Late sodium current inhibition reverses electromechanical dysfunction in human hypertrophic cardiomyopathy. Circulation.

[30] Kuppahally S.S., Akoum N., Burgon N.S., Badger T.J., Kholmovski E.G., Vijayakumar S., Rao S.N., Blauer J., Fish E.N., Dibella E.V. (2010). Left atrial strain and strain rate in patients with paroxysmal and persistent atrial fibrillation: Relationship to left atrial structural remodeling detected by delayed-enhancement MRI. Circ. Cardiovasc. Imaging.

[31] Park K.M., Im S.I., Kim E.K., Lee S.C., Park S.J., Kim J.S., On Y.K. (2016). Atrial Fibrillation in Hypertrophic Cardiomyopathy: Is the Extent of Septal Hypertrophy Important?. PLoS ONE.

[32] Olivotto I., Cecchi F., Casey S.A., Dolara A., Traverse J.H., Maron B.J. (2001). Impact of atrial fibrillation on the clinical course of hypertrophic cardiomyopathy. Circulation.

[33] Masri A., Kanj M., Thamilarasan M., Wazni O., Smedira N.G., Lever H.M., Desai M.Y. (2017). Outcomes in hypertrophic cardiomyopathy patients with and without atrial fibrillation: A survival meta-analysis. Cardiovasc. Diagn. Ther..

[34] Boldt A., Wetzel U., Lauschke J., Weigl J., Gummert J., Hindricks G., Kottkamp H., Dhein S. (2004). Fibrosis in left atrial tissue of patients with atrial fibrillation with and without underlying mitral valve disease. Heart.

[35] Debonnaire P., Joyce E., Hiemstra Y., Mertens B.J., Atsma D.E., Schalij M.J., Bax J.J., Delgado V., Marsan N.A. (2017). Left Atrial Size and Function in Hypertrophic Cardiomyopathy Patients and Risk of New-Onset Atrial Fibrillation. Circ. Arrhythm. Electrophysiol..

[36] Maron B.J., Desai M.Y., Nishimura R.A., Spirito P., Rakowski H., Towbin J.A., Rowin E.J., Maron M.S., Sherrid M.V. (2022). Diagnosis and Evaluation of Hypertrophic Cardiomyopathy: JACC State-of-the-Art Review. J. Am. Coll. Cardiol..

[37] Cannon R.O., Rosing D.R., Maron B.J., Leon M.B., Bonow R.O., Watson R.M., Epstein S.E. (1985). Myocardial ischemia in patients with hypertrophic cardiomyopathy: Contribution of inadequate vasodilator reserve and elevated left ventricular filling pressures. Circulation.

[38] Schlittler M., Pramstaller P.P., Rossini A., De Bortoli M. (2023). Myocardial Fibrosis in Hypertrophic Cardiomyopathy: A Perspective from Fibroblasts. Int. J. Mol. Sci..

[39] Rowin E.J., Maron B.J., Haas T.S., Garberich R.F., Wang W., Link M.S., Maron M.S. (2017). Hypertrophic Cardiomyopathy With Left Ventricular Apical Aneurysm: Implications for Risk Stratification and Management. J. Am. Coll. Cardiol..

[40] Harper A.R., Goel A., Grace C., Thomson K.L., Petersen S.E., Xu X., Waring A., Ormondroyd E., Kramer C.M., Ho C.Y. (2021). Common genetic variants and modifiable risk factors underpin hypertrophic cardiomyopathy susceptibility and expressivity. Nat. Genet..

[41] Massera D., Sherrid M.V., Maron M.S., Rowin E.J., Maron B.J. (2023). How common is hypertrophic cardiomyopathy… really?: Disease prevalence revisited 27 years after CARDIA. Int. J. Cardiol..

[42] Maron B.J., Rowin E.J., Maron M.S. (2018). Global Burden of Hypertrophic Cardiomyopathy. JACC Heart Fail.

[43] Semsarian C., Ingles J., Maron M.S., Maron B.J. (2015). New perspectives on the prevalence of hypertrophic cardiomyopathy. J. Am. Coll. Cardiol..

[44] Siontis K.C., Ommen S.R., Geske J.B. (2019). Sex, Survival, and Cardiomyopathy: Differences Between Men and Women With Hypertrophic Cardiomyopathy. J. Am. Heart Assoc..

[45] Maron B.J., Rowin E.J., Maron M.S. (2022). Hypertrophic Cardiomyopathy: New Concepts and Therapies. Annu. Rev. Med..

[46] Arabadjian M., McCarthy M., Dickson V.V. (2021). An Integrated Review of Hypertrophic Cardiomyopathy in Black Populations: Underrecognized and Understudied. J. Cardiovasc. Nurs..

[47] Maron B.A., Wang R.S., Carnethon M.R., Rowin E.J., Loscalzo J., Maron B.J., Maron M.S. (2022). What Causes Hypertrophic Cardiomyopathy?. Am. J. Cardiol..

[48] Liu G., Su L., Lang M. (2023). A systematic review and meta-analysis of sex differences in clinical outcomes of hypertrophic cardiomyopathy. Front. Cardiovasc. Med..

[49] Lakdawala N.K., Olivotto I., Day S.M., Han L., Ashley E.A., Michels M., Ingles J., Semsarian C., Jacoby D., Jefferies J.L. (2021). Associations Between Female Sex, Sarcomere Variants, and Clinical Outcomes in Hypertrophic Cardiomyopathy. Circ. Genom. Precis Med..

[50] Butters A., Lakdawala N.K., Ingles J. (2021). Sex Differences in Hypertrophic Cardiomyopathy: Interaction With Genetics and Environment. Curr. Heart Fail Rep..

[51] Zaromytidou M., Savvatis K. (2023). The weight of obesity in hypertrophic cardiomyopathy. Clin. Med..

[52] Javaheri S., Barbe F., Campos-Rodriguez F., Dempsey J.A., Khayat R., Javaheri S., Malhotra A., Martinez-Garcia M.A., Mehra R., Pack A.I. (2017). Sleep Apnea: Types, Mechanisms, and Clinical Cardiovascular Consequences. J. Am. Coll. Cardiol..

[53] Jex N., Chowdhary A., Thirunavukarasu S., Procter H., Sengupta A., Natarajan P., Kotha S., Poenar A.M., Swoboda P., Xue H. (2022). Coexistent Diabetes Is Associated with the Presence of Adverse Phenotypic Features in Patients with Hypertrophic Cardiomyopathy. Diabetes Care.

[54] Wu S., Yang L., Sun N., Luo X., Li P., Wang K., Li P., Zhao J., Wang Z., Zhang Q. (2024). Impact of coronary artery disease in patients with hypertrophic cardiomyopathy. Hell. J. Cardiol..

[55] Finocchiaro G., Magavern E., Sinagra G., Ashley E., Papadakis M., Tome-Esteban M., Sharma S., Olivotto I. (2017). Impact of Demographic Features, Lifestyle, and Comorbidities on the Clinical Expression of Hypertrophic Cardiomyopathy. J. Am. Heart Assoc..

[56] Hong Y., Su W.W., Li X. (2022). Risk factors of sudden cardiac death in hypertrophic cardiomyopathy. Curr. Opin. Cardiol..

[57] Maron B.J., McKenna W.J., Danielson G.K., Kappenberger L.J., Kuhn H.J., Seidman C.E., Shah P.M., Spencer W.H., Spirito P., Ten Cate F.J. (2003). American College of Cardiology/European Society of Cardiology clinical expert consensus document on hypertrophic cardiomyopathy. A report of the American College of Cardiology Foundation Task Force on Clinical Expert Consensus Documents and the European Society of Cardiology Committee for Practice Guidelines. J. Am. Coll. Cardiol..

[58] Nagata Y., Kado Y., Onoue T., Otani K., Nakazono A., Otsuji Y., Takeuchi M. (2018). Impact of image quality on reliability of the measurements of left ventricular systolic function and global longitudinal strain in 2D echocardiography. Echo Res. Pract..

[59] Gersh B.J., Maron B.J., Bonow R.O., Dearani J.A., Fifer M.A., Link M.S., Naidu S.S., Nishimura R.A., Ommen S.R., Rakowski H. (2011). 2011 ACCF/AHA Guideline for the Diagnosis and Treatment of Hypertrophic Cardiomyopathy: A report of the American College of Cardiology Foundation/American Heart Association Task Force on Practice Guidelines. Developed in collaboration with the American Association for Thoracic Surgery, American Society of Echocardiography, American Society of Nuclear Cardiology, Heart Failure Society of America, Heart Rhythm Society, Society for Cardiovascular Angiography and Interventions, and Society of Thoracic Surgeons. J. Am. Coll. Cardiol..

[60] Arbelo E., Protonotarios A., Gimeno J.R., Arbustini E., Barriales-Villa R., Basso C., Bezzina C.R., Biagini E., Blom N.A., de Boer R.A. (2023). 2023 ESC Guidelines for the management of cardiomyopathies: Developed by the task force on the management of cardiomyopathies of the European Society of Cardiology (ESC). Eur. Heart J..

[61] Maron B.J., Ommen S.R., Semsarian C., Spirito P., Olivotto I., Maron M.S. (2014). Hypertrophic cardiomyopathy: Present and future, with translation into contemporary cardiovascular medicine. J. Am. Coll. Cardiol..

[62] Cardoso I., Viegas J.M., Rosa S.A., Brás P.G., Grazina A., Cruz I., Branco L.M., Galrinho A., Fiarresga A., Lopes L.R. (2023). Three-dimensional echocardiography for the evaluation of hypertrophic cardiomyopathy patients: Relation to symptoms and exercise capacity. Int. J. Cardiovasc. Imaging.

[63] Erden M., van Velzen H.G., Menting M.E., van den Bosch A.E., Ren B., Michels M., Vletter W.B., van Domburg R.T., Schinkel A.F.L. (2018). Three-dimensional echocardiography for the assessment of left ventricular geometry and papillary muscle morphology in hypertrophic cardiomyopathy. J. Ultrasound..

[64] Andrew C.Y. (2011). To, Ashwat Dhillon, Milind Y. Desai, Cardiac Magnetic Resonance in Hypertrophic Cardiomyopathy. JACC Cardiovasc. Imaging.

[65] Moravsky G., Ofek E., Rakowski H., Butany J., Williams L., Ralph-Edwards A., Wintersperger B.J., Crean A. (2013). Myocardial fibrosis in hypertrophic cardiomyopathy: Accurate reflection of histopathological findings by CMR. JACC Cardiovasc. Imaging.

[66] Noureldin R.A., Liu S., Nacif M.S., Judge D.P., Halushka M.K., Abraham T.P., Ho C., Bluemke D.A. (2012). The diagnosis of hypertrophic cardiomyopathy by cardiovascular magnetic resonance. J. Cardiovasc. Magn. Reson..

[67] Dorobantu D.M., Wadey C.A., Amir N.H., Stuart A.G., Williams C.A., Pieles G.E. (2021). The Role of Speckle Tracking Echocardiography in the Evaluation of Common Inherited Cardiomyopathies in Children and Adolescents: A Systematic Review. Diagnostics.

[68] Quintana R.A., Bui L.P., Moudgil R., Palaskas N., Hassan S., Abe J.I., Mouhayar E., Yusuf S.W., Hernandez A., Banchs J. (2020). Speckle-Tracking Echocardiography in Cardio-Oncology and Beyond. Tex Heart Inst. J..

[69] Biernacka E.K., Osadnik T., Bilińska Z.T., Krawczyński M., Latos-Bieleńska A., Łaczmańska I., Miszczak-Knecht M., Płoski R., Ponińska J.K., Prejbisz A. (2024). A position statement of the Polish Cardiac Society endorsed by Polish Society of Human Genetics and Cardiovascular Patient Communities. Kardiol Pol..

[70] Girolami F., Gozzini A., Pálinkás E.D., Ballerini A., Tomberli A., Baldini K., Marchi A., Zampieri M., Passantino S., Porcedda G. (2023). Genetic Testing and Counselling in Hypertrophic Cardiomyopathy: Frequently Asked Questions. J. Clin. Med..

[71] Tudurachi B.S., Zăvoi A., Leonte A., Țăpoi L., Ureche C., Bîrgoan S.G., Chiuariu T., Anghel L., Radu R., Sascău R.A. (2023). An Update on MYBPC3 Gene Mutation in Hypertrophic Cardiomyopathy. Int. J. Mol. Sci..

[72] Lubitz S.A., Ellinor P.T. (2015). Next-generation sequencing for the diagnosis of cardiac arrhythmia syndromes. Heart Rhythm..

[73] Marian A.J. (2021). Molecular Genetic Basis of Hypertrophic Cardiomyopathy. Circ. Res..

[74] Huurman R., Bowen D.J., Mutluer F.O., Loff Barreto B., van Slegtenhorst M.A., Verhagen J.M.A., Hirsch A., van den Bosch A.E., Michels M., Schinkel A.F.L. (2022). Prognostic significance of left atrial strain in sarcomere gene variant carriers without hypertrophic cardiomyopathy. Echocardiography.

[75] Jansen M., Algül S., Bosman L.P., Michels M., van der Velden J., de Boer R.A., van Tintelen J.P., Asselbergs F.W., Baas A.F. (2022). Blood-based biomarkers for the prediction of hypertrophic cardiomyopathy prognosis: A systematic review and meta-analysis. ESC Heart Fail.

[76] Lim L.J., Tison G.H., Delling F.N. (2020). Artificial Intelligence in Cardiovascular Imaging. Methodist Debakey Cardiovasc. J..

[77] Farahani N.Z., Arunachalam S.P., Sundaram D.S.B., Pasupathy K., Enayati M., Arruda-Olson A.M. Explanatory Analysis of a Machine Learning Model to Identify Hypertrophic Cardiomyopathy Patients from EHR Using Diagnostic Codes. Proceedings of the 2020 IEEE International Conference on Bioinformatics and Biomedicine (BIBM).

[78] Wang Y.-R., Yang K., Wen Y., Wang P., Hu Y., Lai Y., Wang Y., Zhao K., Tang S., Zhang A. (2024). Screening and diagnosis of cardiovascular disease using artificial intelligence-enabled cardiac magnetic resonance imaging. Nat. Med..

[79] Krittanawong C., Johnson K.W., Choi E., Kaplin S., Venner E., Murugan M., Wang Z., Glicksberg B.S., Amos C.I., Schatz M.C. (2022). Artificial Intelligence and Cardiovascular Genetics. Life.

[80] Tower-Rader A., Kramer C.M., Neubauer S., Nagueh S.F., Desai M.Y. (2020). Multimodality Imaging in Hypertrophic Cardiomyopathy for Risk Stratification. Circ. Cardiovasc. Imaging.

[81] Monda E., Palmiero G., Lioncino M., Rubino M., Cirillo A., Fusco A., Caiazza M., Verrillo F., Diana G., Mauriello A. (2022). Multimodality Imaging in Cardiomyopathies with Hypertrophic Phenotypes. J. Clin. Med..

[82] Goldie F.C., Lee M.M.Y., Coats C.J., Nordin S. (2024). Advances in Multi-Modality Imaging in Hypertrophic Cardiomyopathy. J. Clin. Med..

[83] Abraham M.R., Abraham T.P. (2024). Role of Imaging in the Diagnosis, Evaluation, and Management of Hypertrophic Cardiomyopathy. Am. J. Cardiol..

[84] Calderon Martinez E., Ortiz-Garcia N.Y., Herrera Hernandez D.A., Arriaga Escamilla D., Diaz Mendoza D.L., Othon Martinez D., Ramirez L.M., Reyes-Rivera J., Choudhari J., Michel G. (2023). Hypertrophic Cardiomyopathy Diagnosis and Treatment in High- and Low-Income Countries: A Narrative Review. Cureus.

[85] Fernandes F., Antunes M.O., Hotta V.T., Rochitte C.E., Mady C. (2019). Deposit Diseases as Differential Diagnosis of Left Ventricular Hypertrophy in Patients with Heart Failure and Preserved Systolic Function. Arq. Bras. Cardiol..

[86] Ramchand J., Fava A.M., Chetrit M., Desai M.Y. (2020). Advanced imaging for risk stratification of sudden death in hypertrophic cardiomyopathy. Heart.

[87] van der Velden J., Tocchetti C.G., Varricchi G., Bianco A., Sequeira V., Hilfiker-Kleiner D., Hamdani N., Leite-Moreira A.F., Mayr M., Falcão-Pires I. (2018). Metabolic changes in hypertrophic cardiomyopathies: Scientific update from the Working Group of Myocardial Function of the European Society of Cardiology. Cardiovasc. Res..

[88] Gersh B.J., Maron B.J., Dearani J.A., Fifer M.A., Link M.S., Naidu S.S., Nishimura R.A., Ommen S.R., Rakowski H., Seidman C.E. (2011). 2011 ACCF/AHA guideline for the diagnosis and treatment of hypertrophic cardiomyopathy: A report of the American College of Cardiology Foundation/American Heart Association Task Force on Practice Guidelines. Circulation.

[89] Zampieri M., Zampieri M., Berteotti M., Berteotti M., Ferrantini C., Ferrantini C., Tassetti L., Tassetti L., Gabriele M., Gabriele M. (2021). Pathophysiology and Treatment of Hypertrophic Cardiomyopathy: New Perspectives. Curr. Heart Fail. Rep..

[90] Maron B.J. (2018). Clinical Course and Management of Hypertrophic Cardiomyopathy. N. Engl. J. Med..

[91] Dybro A.M., Rasmussen T.B., Nielsen R.R., Andersen M.J., Jensen M.K., Poulsen S.H. (2021). Randomized Trial of Metoprolol in Patients with Obstructive Hypertrophic Cardiomyopathy. J. Am. Coll. Cardiol..

[92] Wigle E.D. (1995). Novel insights into the clinical manifestations and treatment of hypertrophic cardiomyopathy. Curr. Opin. Cardiol..

[93] Ommen S.R., Mital S., Burke M.A., Day S.M., Deswal A., Elliott P., Evanovich L.L., Hung J., Joglar J.A., Kantor P. (2020). 2020 AHA/ACC Guideline for the Diagnosis and Treatment of Patients with Hypertrophic Cardiomyopathy: A Report of the American College of Cardiology/American Heart Association Joint Committee on Clinical Practice Guidelines. J. Am. Coll. Cardiol..

[94] Ho C.Y., Day S.M., Axelsson A., Russell M.W., Zahka K., Lever H.M., Pereira A.C., Colan S.D., Margossian R., Murphy A.M. (2021). Valsartan in early-stage hypertrophic cardiomyopathy: A randomized phase 2 trial. Nat. Med..

[95] Nishimura R.A., Holmes D.R. (2004). Hypertrophic obstructive cardiomyopathy. N. Engl. J. Med..

[96] Nishimura R.A., Seggewiss H., Schaff H.V. (2017). Hypertrophic Obstructive Cardiomyopathy: Surgical Myectomy and Septal Ablation. Circ. Res..

[97] Dearani J.A., Ommen S.R., Gersh B.J., Schaff H.V., Danielson G.K. (2007). Surgery insight: Septal myectomy for obstructive hypertrophic cardiomyopathy--the Mayo Clinic experience. Nature clinical practice. Cardiovasc. Med..

[98] Kimmelstiel C., Zisa D.C., Kuttab J.S., Wells S., Udelson J.E., Wessler B.S., Rastegar H., Kapur N.K., Weintraub A.R., Maron B.J. (2019). Guideline-Based Referral for Septal Reduction Therapy in Obstructive Hypertrophic Cardiomyopathy Is Associated With Excellent Clinical Outcomes. Circulation. Cardiovasc. Interv..

[99] Ottaviani A., Mansour D., Molinari L.V., Galanti K., Mantini C., Khanji M.Y., Chahal A.A., Zimarino M., Renda G., Sciarra L. (2023). Revisiting Diagnosis and Treatment of Hypertrophic Cardiomyopathy: Current Practice and Novel Perspectives. J. Clin. Med..

[100] Mistrulli R., Ferrera A., Salerno L., Vannini F., Guida L., Corradetti S., Addeo L., Valcher S., Di Gioia G., Spera F.R. (2024). Cardiomyopathy and Sudden Cardiac Death: Bridging Clinical Practice with Cutting-Edge Research. Biomedicines.

[101] Losi M.A., Nistri S., Galderisi M., Betocchi S., Cecchi F., Olivotto I., Agricola E., Ballo P., Buralli S., D’Andrea A. (2010). Echocardiography in patients with hypertrophic cardiomyopathy: Usefulness of old and new techniques in the diagnosis and pathophysiological assessment. Cardiovasc. Ultrasound..

[102] Sipola P., Magga J., Husso M., Jääskeläinen P., Peuhkurinen K., Kuusisto J. (2011). Cardiac MRI assessed left ventricular hypertrophy in differentiating hypertensive heart disease from hypertrophic cardiomyopathy attributable to a sarcomeric gene mutation. Eur. Radiol..

[103] De Marco F., Ferrucci F., Risi M., Tortora G. (2022). Classification of QRS complexes to detect Premature Ventricular Contraction using machine learning techniques. PLoS ONE.

[104] De Marco F., Di Biasi L., Auriemma Citarella A., Tortora G., Villani M., Cagnoni S., Serra R. (2024). Improving PVC Detection in ECG Signals: A Recurrent Neural Network Approach. Artificial Life and Evolutionary Computation.

[105] Kujime K., Seno H., Nakajima K., Yamazaki M., Sakuma I., Yamagata K., Kusano K., Tomii N. (2024). Explainable localization of premature ventricular contraction using deep learning-based semantic segmentation of 12-lead electrocardiogram. J. Arrhythm..

[106] Bonaventura J., Polakova E., Vejtasova V., Veselka J. (2021). Genetic Testing in Patients with Hypertrophic Cardiomyopathy. Int. J. Mol. Sci..

[107] Tfelt-Hansen J., Garcia R., Albert C., Merino J., Krahn A., Marijon E., Basso C., Wilde A.A.M., Haugaa K.H. (2023). Risk stratification of sudden cardiac death: A review. Europace.

[108] Vehmeijer J.T., Christiaans I., van Langen I.M., Birnie E., Bonsel G.J., Smets E.M., Wilde A.A. (2009). Risk stratification for sudden cardiac death in hypertrophic cardiomyopathy: Dutch cardiologists and the care of mutation carriers. Neth. Heart J..

[109] Maron M.S., Rowin E.J., Olivotto I., Casey S.A., Arretini A., Tomberli B., Garberich R.F., Link M.S., Chan R.H.M., Lesser J.R. (2016). Contemporary Natural History and Management of Nonobstructive Hypertrophic Cardiomyopathy. J. Am. Coll. Cardiol..

[110] Ferreira P.F., Kilner P.J., McGill L.A., Nielles-Vallespin S., Scott A.D., Ho S.Y., McCarthy K.P., Haba M.M., Ismail T.F., Gatehouse P.D. (2014). In vivo cardiovascular magnetic resonance diffusion tensor imaging shows evidence of abnormal myocardial laminar orientations and mobility in hypertrophic cardiomyopathy. J. Cardiovasc. Magn. Reson..

[111] Bhaltadak V., Ghewade B., Yelne S. (2024). A Comprehensive Review on Advancements in Wearable Technologies: Revolutionizing Cardiovascular Medicine. Cureus.

[112] Abbas M.T., Baba Ali N., Farina J.M., Mahmoud A.K., Pereyra M., Scalia I.G., Kamel M.A., Barry T., Lester S.J., Cannan C.R. (2024). Role of Genetics in Diagnosis and Management of Hypertrophic Cardiomyopathy: A Glimpse into the Future. Biomedicines.

[113] Dong T., Alencherry B., Ospina S., Desai M.Y. (2023). Review of Mavacamten for Obstructive Hypertrophic Cardiomyopathy and Future Directions. Drug Des. Devel Ther..

[114] Zatorski N., Sobie E.A., Schlessinger A. (2023). Mavacamten improves symptoms in obstructive hypertrophic cardiomyopathy patients. Trends Pharmacol. Sci..

[115] Braunwald E., Saberi S., Abraham T.P., Elliott P.M., Olivotto I. (2023). Mavacamten: A first-in-class myosin inhibitor for obstructive hypertrophic cardiomyopathy. Eur. Heart J..

[116] Rangwala H.S., Fatima H., Ali M., Ahmed S.T., Rangwala B.S., Abbas S.R. (2023). Analyzing safety and effectiveness of Mavacamten in comparison with placebo for managing hypertrophic cardiomyopathy: A systemic review and meta-analysis. Egypt Heart J..

[117] Ammirati E., Gallone G. (2024). Mavacamten: Practical Answers for the Clinician and New Questions From the MAVA-Long-Term Extension Study. JACC Heart Fail.

[118] Woodland M., Al-Horani R.A. (2023). New Era: Mavacamten for Obstructive Hypertrophic Cardiomyopathy. Cardiovasc. Hematol. Agents Med. Chem..

[119] Keam S.J. (2022). Mavacamten: First Approval. Drugs.

[120] Bello J., Pellegrini M.V. (2024). Mavacamten. 2024 Aug 21. StatPearls [Internet].

[121] Nag S., Gollapudi S.K., Del Rio C.L., Spudich J.A., McDowell R. (2023). Mavacamten, a precision medicine for hypertrophic cardiomyopathy: From a motor protein to patients. Sci. Adv..

[122] Ismayl M., Abbasi M.A., Marar R., Geske J.B., Gersh B.J., Anavekar N.S. (2023). Mavacamten Treatment for Hypertrophic Cardiomyopathy: A Systematic Review and Meta-Analysis of Randomized Controlled Trials. Curr. Probl. Cardiol..

[123] Zampieri M., Argirò A., Marchi A., Berteotti M., Targetti M., Fornaro A., Tomberli A., Stefàno P., Marchionni N., Olivotto I. (2021). Mavacamten, a Novel Therapeutic Strategy for Obstructive Hypertrophic Cardiomyopathy. Curr. Cardiol. Rep..

[124] Aguiar T., Martins E. (2022). Mavacamten, a novel revolutionizing therapy in hypertrophic obstructive cardiomyopathy: A literature review. Rev. Port. Cardiol..

[125] Wheeler M.T., Jacoby D., Elliott P.M., Saberi S., Hegde S.M., Lakdawala N.K., Myers J., Sehnert A.J., Edelberg J.M., Li W. (2023). Effect of beta-blocker therapy on the response to mavacamten in patients with symptomatic obstructive hypertrophic cardiomyopathy. Eur. J. Heart Fail.

[126] Flenner F., Geertz B., Reischmann-Düsener S., Weinberger F., Eschenhagen T., Carrier L., Friedrich F.W. (2017). Diltiazem prevents stress-induced contractile deficits in cardiomyocytes, but does not reverse the cardiomyopathy phenotype in Mybpc3-knock-in mice. J. Physiol..

[127] Dominguez F., Cabrera E. (2023). Mavacamten in obstructive hypertrophic cardiomyopathy—Are beta-blockers blocking part of its shine?. Eur. J. Heart Fail.

[128] Bishev D., Fabara S., Loseke I., Alok A., Al-Ani H., Bazikian Y. (2023). Efficacy and Safety of Mavacamten in the Treatment of Hypertrophic Cardiomyopathy: A Systematic Review. Heart Lung Circ..

[129] Rabiee Rad M., Ghasempour Dabaghi G., Habibi D. (2023). Safety and efficacy of mavacamten for treatment of hypertrophic cardiomyopathy: A systematic review and meta-analysis of randomized clinical trials. Egypt Heart J..

[130] Olivotto I., Oreziak A., Barriales-Villa R., Abraham T.P., Masri A., Garcia-Pavia P., Saberi S., Lakdawala N.K., Wheeler M.T., Owens A. (2020). Mavacamten for treatment of symptomatic obstructive hypertrophic cardiomyopathy (EXPLORER-HCM): A randomised, double-blind, placebo-controlled, phase 3 trial. Lancet.

[131] Rader F., Oręziak A., Choudhury L., Saberi S., Fermin D., Wheeler M.T., Abraham T.P., Garcia-Pavia P., Zwas D.R., Masri A. (2024). Mavacamten Treatment for Symptomatic Obstructive Hypertrophic Cardiomyopathy: Interim Results From the MAVA-LTE Study, EXPLORER-LTE Cohort. JACC Heart Fail.

[132] Ho C.Y., Olivotto I., Jacoby D., Lester S.J., Roe M., Wang A., Waldman C.B., Zhang D., Sehnert A.J., Heitner S.B. (2020). Study Design and Rationale of EXPLORER-HCM: Evaluation of Mavacamten in Adults with Symptomatic Obstructive Hypertrophic Cardiomyopathy. Circ. Heart Fail.

[133] Spertus J.A., Fine J.T., Elliott P., Ho C.Y., Olivotto I., Saberi S., Li W., Dolan C., Reaney M., Sehnert A.J. (2021). Mavacamten for treatment of symptomatic obstructive hypertrophic cardiomyopathy (EXPLORER-HCM): Health status analysis of a randomised, double-blind, placebo-controlled, phase 3 trial. Lancet.

[134] Desai M.Y., Owens A., Wolski K., Geske J.B., Saberi S., Wang A., Sherrid M., Cremer P.C., Lakdawala N.K., Tower-Rader A. (2023). Mavacamten in Patients with Hypertrophic Cardiomyopathy Referred for Septal Reduction: Week 56 Results From the VALOR-HCM Randomized Clinical Trial. JAMA Cardiol..

[135] Desai M.Y., Owens A., Geske J.B., Wolski K., Naidu S.S., Smedira N.G., Cremer P.C., Schaff H., McErlean E., Sewell C. (2022). Myosin Inhibition in Patients with Obstructive Hypertrophic Cardiomyopathy Referred for Septal Reduction Therapy. J. Am. Coll. Cardiol..

[136] Cremer P.C., Geske J.B., Owens A., Jaber W.A., Harb S.C., Saberi S., Wang A., Sherrid M., Naidu S.S., Schaff H. (2022). Myosin Inhibition and Left Ventricular Diastolic Function in Patients with Obstructive Hypertrophic Cardiomyopathy Referred for Septal Reduction Therapy: Insights From the VALOR-HCM Study. Circ. Cardiovasc. Imaging.

[137] Desai M.Y., Okushi Y., Wolski K., Geske J.B., Owens A., Saberi S., Wang A., Cremer P.C., Sherrid M., Lakdawala N.K. (2024). Mavacamten-Associated Temporal Changes in Left Atrial Function in Obstructive HCM: Insights from the VALOR-HCM Trial. JACC Cardiovasc. Imaging.

[138] Desai M.Y., Owens A., Geske J.B., Wolski K., Saberi S., Wang A., Sherrid M., Cremer P.C., Naidu S.S., Smedira N.G. (2023). Dose-Blinded Myosin Inhibition in Patients With Obstructive Hypertrophic Cardiomyopathy Referred for Septal Reduction Therapy: Outcomes Through 32 Weeks. Circulation.

[139] Wilcox J.E., McNally E.M. (2020). Lessons From MAVERICK-HCM: The Need for Less Speed. J. Am. Coll. Cardiol..

[140] Ho C.Y., Mealiffe M.E., Bach R.G., Bhattacharya M., Choudhury L., Edelberg J.M., Hegde S.M., Jacoby D., Lakdawala N.K., Lester S.J. (2020). Evaluation of Mavacamten in Symptomatic Patients With Nonobstructive Hypertrophic Cardiomyopathy. J. Am. Coll. Cardiol..

[141] Liao H.L., Liang Y., Liang B. (2024). Evaluation of mavacamten in patients with hypertrophic cardiomyopathy. J. Cardiovasc. Med..

[142] Scholtz S., Rudolph V., Reil J.C. (2023). Alcohol Septal Ablation or Mavacamten for Obstructive Hypertrophic Cardiomyopathy. J. Clin. Med..

[143] Garcia-Pavia P., Oręziak A., Masri A., Barriales-Villa R., Abraham T.P., Owens A.T., Jensen M.K., Wojakowski W., Seidler T., Hagege A. (2024). Long-term effect of mavacamten in obstructive hypertrophic cardiomyopathy. Eur. Heart J..

[144] Masri A., Lester S.J., Stendahl J.C., Hegde S.M., Sehnert A.J., Balaratnam G., Shah A., Fox S., Wang A. (2024). Long-Term Safety and Efficacy of Mavacamten in Symptomatic Obstructive Hypertrophic Cardiomyopathy: Interim Results of the PIONEER-OLE Study. J. Am. Heart Assoc..

[145] Tuohy C.V., Kaul S., Song H.K., Nazer B., Heitner S.B. (2020). Hypertrophic cardiomyopathy: The future of treatment. Eur. J. Heart Fail.

